# Fabrication and Photocatalytic Properties of Electrospun Fe-Doped TiO_2_ Nanofibers Using Polyvinyl Pyrrolidone Precursors

**DOI:** 10.3390/polym13162634

**Published:** 2021-08-07

**Authors:** Kyeong-Han Na, Bo-Sung Kim, Han-Sol Yoon, Tae-Hyeob Song, Sung-Wook Kim, Churl-Hee Cho, Won-Youl Choi

**Affiliations:** 1 Department of Advanced Materials Engineering, Gangneung-Wonju National University, Gangneung 25457, Korea; nag0717@naver.com (K.-H.N.); kbs225@naver.com (B.-S.K.); ac.hsyoon@gmail.com (H.-S.Y.); 2 Korea Institute of Civil Engineering and Building Technology, Goyang 10223, Korea; thsong@kict.re.kr (T.-H.S.); swkim@kict.re.kr (S.-W.K.); 3 Graduate School of Energy Science and Technology, Chungnam National University, Daejeon 34134, Korea; 4 Research Institute for Dental Engineering, Gangneung-Wonju National University, Gangneung 25457, Korea

**Keywords:** Fe-doping, TiO_2_ nanofibers, electrospinning, photocatalyst, photodegradation

## Abstract

For the removal of pollutants, a modified TiO_2_ photocatalyst is attracting attention. Fe-doped TiO_2_ nanofibers were prepared through a combination of electrospinning and calcination. Morphological characterization of the sample was conducted using field-emission scanning electron and transmission electron microscopy. The crystal structure of each sample was analyzed using high-resolution transmission electron microscopy, selected area electron diffraction, and Fast Fourier Transform imaging. The average diameter of the Fe-doped TiO_2_ nanofibers was measured to be 161.5 nm and that of the pure TiO_2_ nanofibers was 181.5 nm. The crystal phase when heat treated at 350 °C was anatase for TiO_2_ nanofibers and rutile for Fe-doped TiO_2_ nanofibers. The crystal phase of the TiO_2_ matrix was easily transitioned to rutile by Fe-doping. The photocatalytic performance of each sample was compared via the photodegradation of methylene blue and acid orange 7 under ultraviolet and visible light irradiation. In the Fe-doped TiO_2_ nanofibers, photodegradation rates of 38.3% and 27.9% were measured under UV irradiation and visible light, respectively. Although other catalysts were not activated, the photodegradation rate in the Fe-doped TiO_2_ nanofibers was 9.6% using acid orange 7 and visible light. For improved photocatalytic activity, it is necessary to study the concentration control of the Fe dopant.

## 1. Introduction

Since Honda and Fujishima reported photoelectrolysis photoelectrodes without an external power source in 1972 [1], TiO_2_ has been drawing substantial attention, and many studies have been conducted to apply TiO_2_ to various industrial fields, such as sensors [2,3,4,5], drug delivery systems [6,7,8], photocatalysts [9,10,11,12], and photoelectrodes [13,14,15]. TiO_2_ has many attractive properties; among them, non-toxicity, strong durability, and excellent chemical stability are regarded as suitable photocatalyst properties for water purification. Despite these excellent properties of TiO_2_, some issues must be addressed to ensure its applicability in industry: electron–hole pair recombination and electromagnetic wave absorption bands that are too wide. In order to accomplish the reduction–oxidation cycle that is targeted for applications as a photocatalyst, electrons in the valence band are excited by absorbing electromagnetic waves that are incident from the outside, and the excited electrons and holes must then be transferred to the surface of the semiconductor to react with the pollution. However, most of the excited electron-hole pairs are recombined in the transfer process to the surface, and the electrical energy is converted to heat energy. In this case, electrons and holes cannot contribute to the photocatalytic reaction. Second, there is the issue of the electromagnetic wave absorption band. The wide bandgap due to the unique electronic structure of the TiO_2_ gives strong oxidizing power to photoelectrons, but at the same time, it increases the light energy required to generate an excited electron–hole pair.

For this reason, in the case of anatase TiO_2_ with a bandgap of 3.2 eV, only a short wavelength no longer than 387.5 nm can be used for the reaction, which means that 95% of the sunlight incident on the earth cannot be used. Because these factors directly reduce the efficiency of the photocatalyst, various methods have been proposed to solve these issues, including elemental doping [16,17,18,19,20,21,22,23], dye sensitization [24,25,26], and microstructure control [27,28,29]. In the case of elemental doping, the dopant acts as a trap for excited electrons or holes, which delays the recombination of electron–hole pairs. Trap states due to the dopant energy level effectively separate the electron–hole pairs and their redox sites. However, at excessively high dopant concentrations, it should be noted that the photoelectric conversion efficiency may be reduced because of competition with the carrier transfer process at the surface. At the same time, if an appropriate element is doped, the band of the wavelength of the required electromagnetic wave can be controlled by improving the electronic structure. The dopant energy level in the TiO_2_ band gap expands the range of usable light energy [30,31,32]. Since elemental doping is an effective solution that can address both of the issues mentioned above, it is important to determine the appropriate element and optimized concentration. Commonly considered appropriate doping elements are metals [33,34,35,36] such as Cu, Fe, Mo, Ni; noble metals [37,38,39] such as Au, Ag, Pd; and anions [40,41,42,43,44] such as N, C, B, P, and S.

In addition, studies to optimize photoelectric conversion and transfer efficiency through morphology and specific surface area control have been conducted extensively, and various structures and synthesis methods have been suggested. To fabricate various TiO_2_ nanostructures such as nanoparticles, nanorods, nanotubes, nanograins, and nanowires, some synthesis methods have been considered, such as hydrothermal methods, sol–gel methods, chemical vapor deposition (CVD), anodization, microwave synthesis, and electrospinning [45,46,47,48,49]. Furthermore, efficiency can be improved by optimizing the shape anisotropy and the electrical properties of each microstructure. 

Among them, electrospinning is a process using the jet spraying and the stretching of a polymer solution generated by an electrostatic force and is an effective process to fabricate one dimensional nanofibers. Although the basic ideas of electrohydrodynamics (EHD) have been explored since around the 16th century, earnest studies to apply them to microfiber fabrication have been discussed by researchers such as Williams and Taylor, starting with the patent of A. Formhals in 1934. Recently, various structures such as homogeneous [50], core-shell [51], Janus [52], tri-layer core–shell [53], and other complicated [54] nanofibers can be fabricated based on a basic understanding and process modeling. To use these various structures effectively, many studies have been conducted such as a biocompatible drug delivery system with antibacterial activity [55,56,57,58,59]. Additionally, various types of structures using nanofibers can be fabricated through after-treatment methods such as calcination [60] and cross-linking [61,62,63].

In this study, we used an electrospinning process to effectively fabricate TiO_2_ semiconductor nanofibers. Iron nitrate was added to a typical precursor solution for fabricating TiO_2_ nanofibers to dope the Fe element to the nanofibers. We anticipated that the fibrous nanofibers fabricated using the electrospinning process to improve mobility by limiting the pathway of the charge carriers to be one-dimensional and by suppressing recombination. At the same time, we reduced the required cost and time through an integrated fabrication process and nanostructure doping. The crystal structure and morphology of the calcined nanofibers were observed using a field emission scanning electron microscope (FE-SEM), a transmission electron microscope (TEM), and energy dispersive X-ray spectroscopy (EDS). A photo-degradation test was conducted using various photocatalysts, methylene blue aqueous solution, and an ultraviolet light source.

## 2. Materials and Methods

### 2.1. Chemicals

The reagents used to prepare the precursor solutions were as follows: titanium tetraisopropoxide (TTIP, ≥98.0%, GR, Junsei, Tokyo, Japan), acetyl acetone (ACAC, ≥99.0%, GR, Junsei, Japan), ethyl alcohol (EtOH, ≥99.5% EP, Daejung, Siheung, Korea), polyvinyl pyrrolidone (PVP, M.W. 1,300,000, Alfa Aesar, Haverhill, MA, USA), Fe(NO_3_)_2_·9H_2_O (GR, ≥ 99.0%, Kanto Chemical, Japan), titanium(IV) oxide (P25, ≥95.0%, Sigma-Aldrich, St. Louis, MO, USA), methylene blue ( ≥82.0%, Sigma-Aldrich, St. Louis, MO, USA), and acid-orange 7(AO7, ≥85.0%, Acros organics, Suwanee, GA, USA).

### 2.2. Electrospinning Process

First, 10.0 g of PVP was added to 60.0 g of EtOH and was stirred for 24 h using a magnetic stirrer to prepare a polymer solution. In another beaker, an aqueous solution of 7.5 g of TTIP, 10.0 g of ACAC, 2.5 g of Fe(NO_3_)_2_·9H_2_O, and 10.0 g of deionized water was stirred for 4 h. The polymer solution and the aqueous solution were then mixed and stirred for 2 h to prepare a reddish-brown precursor solution. In order to compare the photocatalytic activity caused by the Fe-doping, a precursor solution for the pure TiO_2_ nanofibers was prepared by only removing the Fe(NO_3_)_2_·9H_2_O from the same composition. 

The two prepared precursor solutions were loaded into a polypropylene syringe with a diameter of 15.56 mm and a volume of 12 mL and were then mounted on a syringe pump. The syringes were connected to a stainless steel adapter and a 23-gauge needle using polypropylene tubing. We subsequently applied high voltage to the nozzle adapter using a power supplier and a constant flow rate at the same time to induce jet spinning from the droplets of the solution to the grounded aluminum foil collector. The applied electrospinning conditions were as follows: distance between electrodes of 20 cm, an applied voltage of 20 kV, a flow rate of 1.0 mL/h, room temperature, and humidity less than 40%. The composite nanofibers collected on the aluminum foil during the electrospinning process were collected every 1 h and were dried at 60 °C in a dry oven. The calcination process was then conducted at 350 °C using a box furnace. The temperature ramping speed was 5 °C/min.

### 2.3. Characterization

The crystal structure and morphology of the calcined nanofibers were analyzed by a field emission scanning electron microscope (FE-SEM, Inspect F, FEI Korea Co., Ltd., Gyeonggi, Korea), a transmission electron microscope (TEM, JEM-2200FS, Jeol Co., Ltd., Tokyo, Japan), and energy dispersive X-ray spectroscopy (EDS). In addition, a photodegradation test was conducted using a photocatalyst/methylene blue aqueous solution and an ultraviolet light source, and the degradation rate was compared using an ultraviolet–visible spectrophotometer (UV–Vis, G1103A, Agilent Co., Ltd., Santa Clara, CA, USA). To determine the calcination temperature, Thermogravimetric analysis (TGA) was conducted using a thermogravimetric analyzer (TGA, Q500, TA instruments, New Castle, DE, USA).

### 2.4. Photocatalytic Degradation Test

All steps of the photocatalytic degradation tests were conducted in a condition where the natural sunlight was blocked. Methylene blue, AO7, and distilled water were used to simulate contaminated water. Since it was necessary to apply the same contamination concentration of all test samples, 5 mg/L and 20 mg/L of methylene blue and AO7 aqueous solutions, respectively, were prepared in large-capacity bottles. As catalysts for photodegradation, P25, TiO_2_ nanofibers, and Fe-doped TiO_2_ nanofibers were used. To fabricate the mixture samples, 0.2 g of each catalyst and 20 mL of deionized water were added to a Pyrex beaker and were stirred for 30 min. Subsequently, 200 mL of methylene blue and AO7 aqueous solution were added. The beaker was then wrapped with aluminum foil to completely block the incident light, and the mixture was stirred for 2 h. A photocatalytic degradation test of each mixture was conducted in a darkroom using UV (6W, 365 nm) and Vis (500 W, Xenon lamp) light sources for 3 h. During degradation, the catalyst filtered solution was sampled by using a syringe and a polyvinylidene fluoride syringe filter (PVDF filter, 0.2 μm, Whatman, Marlborough, MA, USA) every 30 min and was stored in a cuvette wrapped in aluminum foil. The irradiation distance between the light source and the sample was fixed at 10 cm, and the rotation speed of the stirrer was 240 rpm.

## 3. Results and Discussion

**Figure 1:**
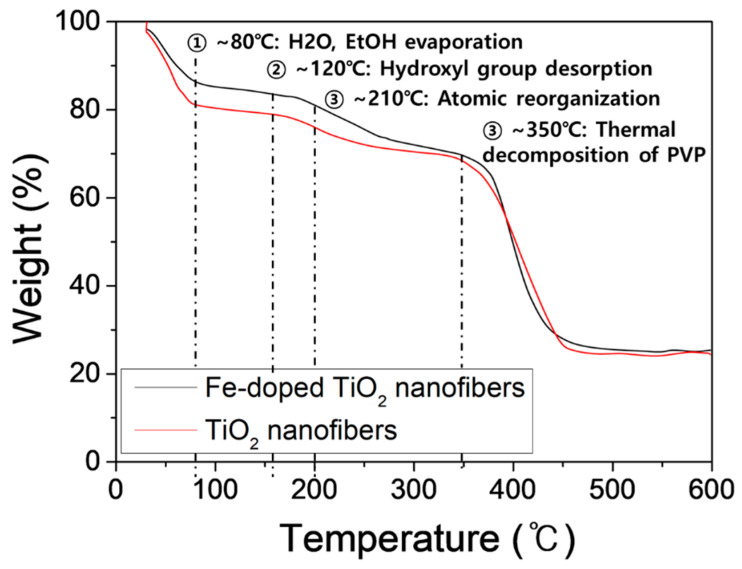
TGA curves of Fe-doped TiO_2_ nanofibers and TiO_2_ nanofibers.

To determine the calcination temperature, TGA was conducted. Figure 1 shows the TGA curves of the TiO_2_ nanofibers and the Fe-doped TiO_2_ nanofibers. N_2_ was used as a purge gas for analysis, and the ramping speed was 5 °C/min. Both samples showed similar thermal decomposition behavior. Until the temperature in the chamber reached 120 °C, the moisture and the solvents adsorbed on the samples evaporated, and the weight decreased. Crystallization started at 210 °C, and the carbonization and the thermal decomposition of the PVP started near 350 °C, causing rapid weight loss. Since N_2_ was used as the purge gas, it showed thermal decomposition behavior and not combustion. The final weights of the TiO_2_ and the Fe-doped TiO_2_ nanofibers were 20.3 wt % and 22.3 wt %, respectively. The difference was due to the non-volatile components that increased with the addition of Fe nitrate in the same precursor solution.

**Figure 2:**
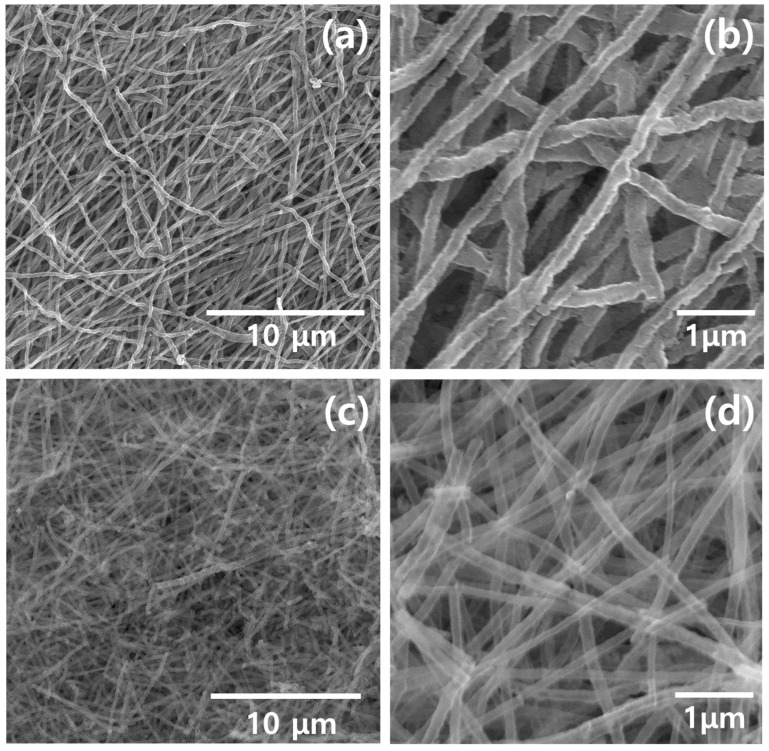
Field-emission scanning electron microscope images of nanofibers: (**a**) low-magnification images of the TiO_2_ nanofiber, (**b**) high-magnification images of the TiO_2_ nanofiber, (**c**) low-magnification images of the Fe-doped TiO_2_ nanofiber, and (**d**) high-magnification images of the Fe-doped TiO_2_ nanofiber.

Figure 2 shows FE-SEM images obtained from the TiO_2_ and Fe-doped TiO_2_ samples. Figure 2a,b are low and high magnification images of the TiO_2_ nanofibers, and c and d are images of the Fe-doped TiO_2_ nanofibers, respectively. It was confirmed that uniform fibrous structures were obtained, thus reflecting that the electrospinning process and calcination were conducted using appropriate conditions in which defects such as cracks or beads did not form.

**Figure 3:**
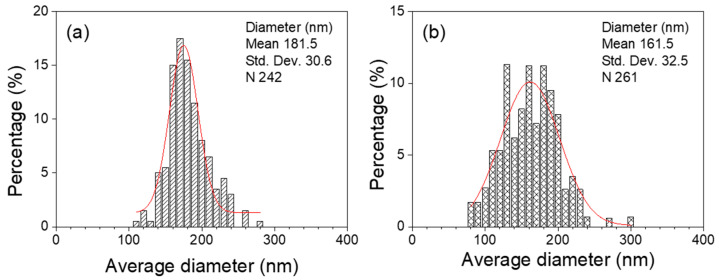
Diameter distribution histogram of nanofibers: (**a**) TiO_2_ nanofibers and (**b**) Fe-doped TiO_2_ nanofibers.

The average diameters of the nanofibers were measured in the obtained FE-SEM images. Figure 3 presents the diameter histogram of the nanofibers. The average diameters of the TiO_2_ and Fe-doped TiO_2_ nanofibers were measured to be 181.5 nm and 162.5 nm, respectively. The content of non-volatile components remaining after the calcination was higher in the Fe-doped TiO_2_ nanofibers, but the average diameter was lower than that of the TiO_2_ nanofibers. We considered that the stretching process was more vigorous because the dielectric constant of the solution was increased due to the added Fe(NO_3_)_2_·9H_2_O.

**Figure 4:**
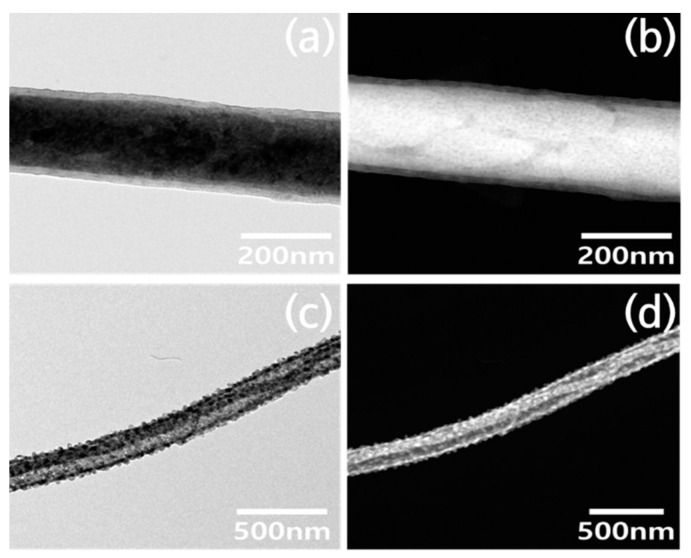
TEM images of nanofibers obtained from each precursor solution: (**a**) bright-field images (BF) of the TiO_2_ nanofiber, (**b**) high-angle annular dark-field images (HAADF) of the TiO_2_ nanofiber, (**c**) BF images of the Fe-doped TiO_2_ nanofiber, and (**d**) HAADF images of the Fe-doped TiO_2_ nanofiber.

Images of the morphology of a single nanofiber were obtained using TEM. Figure 4a,b show bright-field images (BF) and high-angle annular dark-field images (HADDF) of the TiO_2_ nanofibers, respectively. Figure 4c,d are TEM images of Fe-doped TiO_2_ nanofibers. It can be seen that the inside and surface of the TiO_2_ nanofiber have a uniform single phase, but there are many particles of another phase on the Fe-doped TiO_2_ nanofibers. The diameters of the particles formed on the Fe-doped TiO_2_ nanofibers are distributed in the range of 23–29 nm. The reason for this morphological difference was confirmed in other analysis results using TEM accessories.

**Figure 5:**
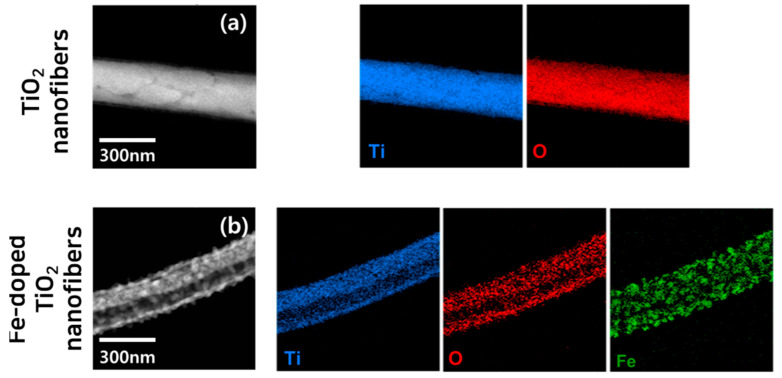
EDS map data of each nanofiber: (**a**) TiO_2_ nanofibers and (**b**) Fe-TiO_2_ nanofibers.

Figure 5 shows the EDS mapping results of a single nanofiber sample. In both samples, titanium and oxygen are uniformly distributed throughout the whole nanofiber, but it can be seen that Fe was mainly located in another phase site that was discontinuously distributed on the Fe-doped TiO_2_ nanofiber. This means that the Fe dopant did not diffuse into the TiO_2_ lattice as an interstitial or substitutional atom during crystal growth and did not form a secondary phase. In order to quantify the ratio of each element, an additional EDS spectrum analysis using FE-SEM was conducted for the Fe-doped TiO_2_ nanofibers. The atomic ratios of Ti, O, and Fe were 29.18 at%, 63.45 at%, and 7.37 at%, respectively, which means that the TiO_2_ and gamma-ferrite phases were well separated and heterogeneous, as confirmed in the TEM image.

**Figure 6:**
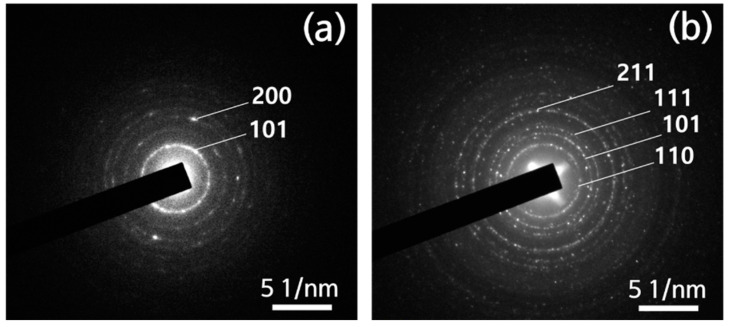
Selected area electron diffraction patterns obtained from nanofibers: (**a**) TiO_2_ nanofibers and (**b**) Fe-doped TiO_2_ nanofibers.

Selected area electron diffraction results for each fiber are shown in Figure 6a,b. Both Figure 6a,b show a ring-type diffraction pattern rather than a circle. This means that both samples were well crystallized. In the case of the amorphous phase, the pattern is diffused and shows a circular pattern. Figure 6a shows the diffraction pattern of the TiO_2_ nanofiber, and the (101) plane, which has a significant peak of the anatase phase, was identified. On the other hand, Figure 6b shows the diffraction pattern of the Fe-doped TiO_2_ nanofiber, and it was indexed to the rutile phase. In general, when the heat treatment temperature increased, the anatase phase transitions to rutile and a high-temperature stable phase, but the calcination temperature of the two samples was the same at 350 °C. This result is the same as in a previous study where Fe doping reduced the high-temperature stability of TiO_2_ and prompted the transition to the rutile phase [64].

**Figure 7:**
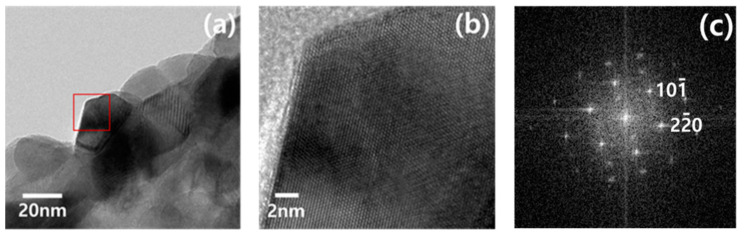
High-resolution transmission electron microscopy (HRTEM) analysis of particles on the surface of Fe-doped TiO_2_ nanofibers: (**a**) BF image of the particle, (**b**) HRTEM image of the particle, and (**c**) FFT patterns of the particle.

Figure 7 shows the high-resolution transmission electron microscopy (HRTEM) images and the FFT pattern of the secondary phase particles on the Fe-doped TiO_2_ nanofibers. The analysis results reveal that the phase of the doped particles is γ-ferrite (austenite) and that it has a face-centered cubic structure.

**Figure 8:**
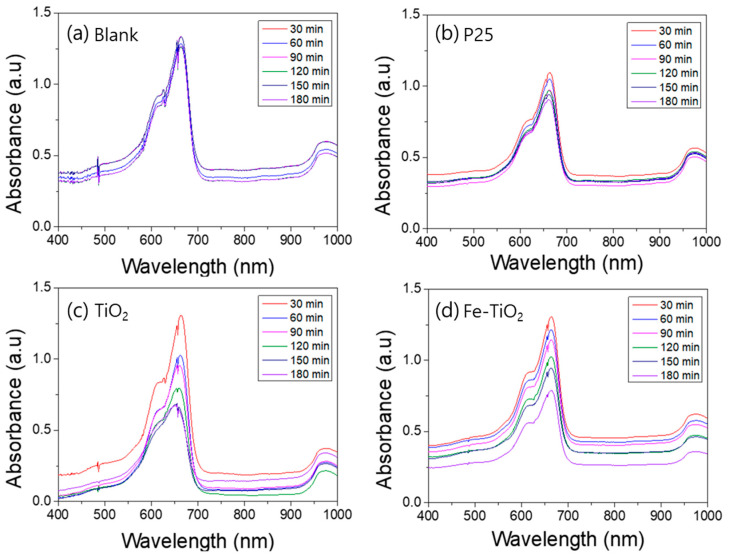
UV–Vis absorption spectra of the ultraviolet photocatalytic degradation of an aqueous methylene blue solution by nanofibers: (**a**) blank, (**b**) P25, (**c**) TiO_2_ nanofibers, (**d**) Fe-doped TiO_2_ nanofibers.

**Figure 9:**
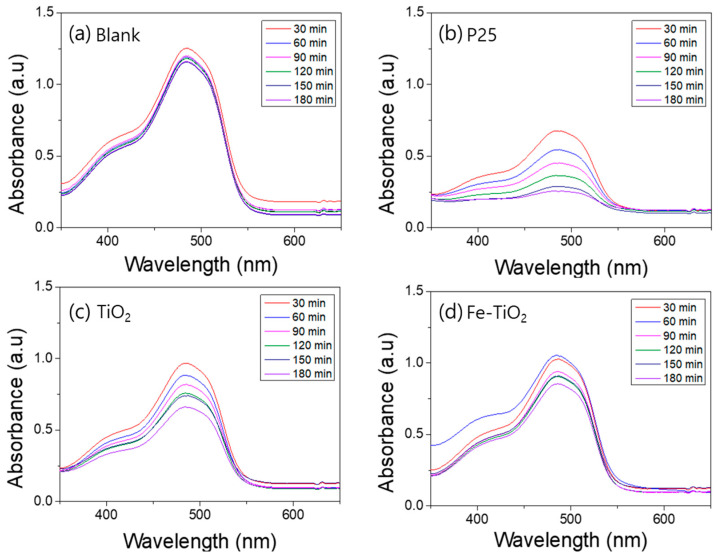
UV–Vis absorption spectra of the ultraviolet photocatalytic degradation of an aqueous acid orange 7 solution by nanofibers: (**a**) blank, (**b**) P25, (**c**) TiO_2_ nanofibers, (**d**) Fe-doped TiO_2_ nanofibers.

**Figure 10:**
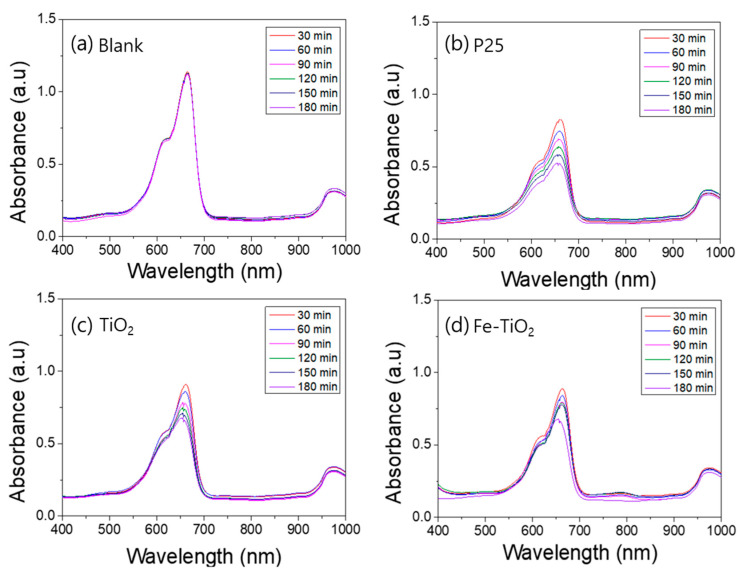
UV–Vis absorption spectra of the visible light photocatalytic degradation of an aqueous methylene blue solution by nanofibers: (**a**) blank, (**b**) P25, (**c**) TiO_2_ nanofibers, (**d**) Fe-doped TiO_2_ nanofibers.

**Figure 11:**
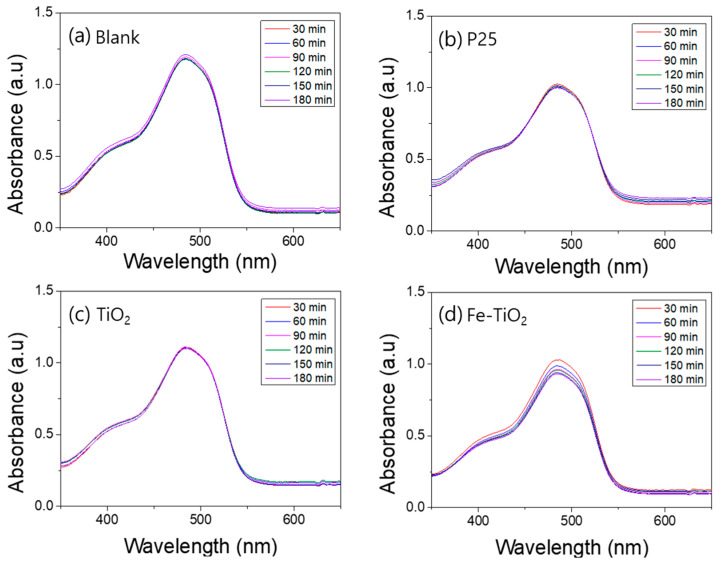
UV–Vis absorption spectra of the visible light photocatalytic degradation of an aqueous acid orange 7 solution by nanofibers: (**a**) blank, (**b**) P25, (**c**) TiO_2_ nanofibers, (**d**) Fe-doped TiO_2_ nanofibers.

A photodegradation test was conducted to compare the photocatalytic activity of the TiO_2_ nanofibers and the Fe-doped TiO_2_ nanofibers. Figure 8, Figure 9, Figure 10 and Figure 11 show the UV–Vis absorbance of various aqueous solutions sampled every 30 min. Figure 7a plots the absorption spectra of the UV exposed pure methylene blue solution without a photocatalyst, and b, c, and d plot the spectra of the mixture containing P25, TiO_2_ nanofibers, and Fe-doped TiO_2_ nanofibers, respectively. Figure 9 shows that AO7 is used as a dye instead of methylene blue, and Figure 10 and Figure 11 show the samples where a Xenon lamp was used as a light source instead of UV. As shown in the graphs, dye degradation was not observed in the blank solution, but it was confirmed that the absorbances of the solutions that dispersed each nanofiber and P25 significantly decreased over time. However, when comparing the degradation tendency at the wavelengths of 663 nm and 483 nm, corresponding with the significant absorption edge of methylene blue and AO7, it can be seen that the photocatalytic performance of the TiO_2_ nanofibers in the anatase phase is slightly superior to that of Fe-doped TiO_2_ nanofibers with the rutile phase. The photocatalytic degradation performance was affected by the crystal phase and metal ion doping, and the effect of the anatase phase was more dominant than the effect of metal ion doping. The photocatalytic degradation performance of P25 was also observed to be superior to that of the nanofibers since P25 has a larger surface area than the TiO_2_ and the Fe-doped TiO_2_ nanofibers.

**Figure 12:**
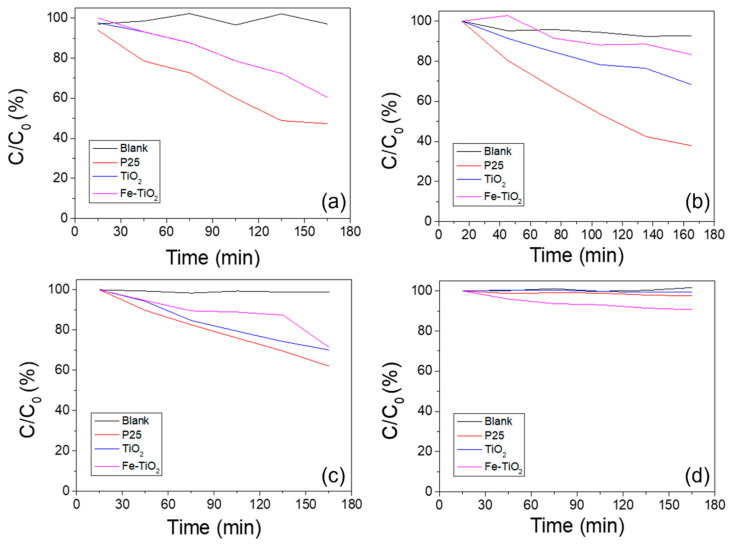
Photodegradation rate of methylene blue and acid orange 7 by various photocatalysts and light sources: (**a**) ultraviolet and methylene blue, (**b**) ultraviolet and acid orange 7, (**c**) visible light and methylene blue, (**d**) visible light and acid orange 7.

Figure 12 shows the photodegradation rate obtained by the absorbance changes of various catalysts every 30 min at the absorption edge. As shown in Figure 8, Figure 9, Figure 10 and Figure 11, there was no change in the blank sample. In the Fe-doped TiO_2_ nanofibers, photodegradation rates of 38.3% and 27.9% were measured under UV irradiation and visible light, respectively. In both the acid AO7 and the methylene blue, the Fe-doped TiO_2_ showed lower photodegradation performance than the pure TiO_2_ photocatalysts, but the difference was decreased under visible light irradiation. Although other catalysts were not activated, the photodegradation rate in Fe-doped TiO_2_ nanofibers was 9.6% when using AO7 and visible light. We considered two reasons for this tendency. The first is the difference in photocatalytic activity due to the TiO_2_ crystal structure. As confirmed by the TEM analysis, the phase of the TiO_2_ matrix in the Fe-doped TiO_2_ is rutile, and previous studies proved that the rutile phase has lower photocatalytic performance compared to the anatase phase [65,66,67]. The second reason is an excessive dopant concentration. If the concentration of the dopant is too high, the photocatalytic efficiency can be suppressed because electron trapping competes with the surface transfer reaction and reduces the sites on the TiO_2_ surface where the holes can react with pollution. As seen in Figure 12d, for the case using visible light and acid orange 7, although other catalysts were not activated, the photodegradation rate in the Fe-doped TiO_2_ nanofibers was 9.6%. The band gap narrowed by Fe-doping increased the photocatalytic performance under visible light. Further research is needed to confirm the special benefits of the narrow band electron structure of Fe-doped TiO_2_ nanofibers in low-energy incident light and azo dyes.

## 4. Conclusions

TiO_2_ is an important material that is being discussed for the removal of organic pollutants. To solve the low photoelectric conversion efficiency issue, we considered Fe-doping among various elements. TiO_2_ and Fe-doped TiO_2_ nanofibers were fabricated by an electrospinning process. Fe-doped TiO_2_ nanofibers were obtained in a single step by adding a dopant element to a solution in a precursor. The average diameter of the nanofibers electrospun with the proposed precursor solution composition was measured to be 181.5 nm for TiO_2_ and 162.5 nm for Fe-doped TiO_2_. When heat treated at 350 °C, the crystal phase was anatase for the TiO_2_ nanofibers and rutile for the Fe-doped TiO_2_ nanofibers. The Fe-doped TiO_2_ nanofibers showed lower photocatalytic performance compared to the TiO_2_ nanofibers because of the rutile crystal phase and excessive Fe concentration. The photocatalytic degradation performance was mainly influenced by the crystal phase and the metal ion doping, and the effect of the anatase phase was more dominant than the effect of the metal ion doping. The photodegradation rate of 9.6% in Fe-doped TiO_2_ nanofibers using visible light suggests a research direction for photocatalytic materials for environmental applications.

## Data Availability

The data supporting the findings of this manuscript are available from the corresponding authors upon reasonable request.

## References

[1] Fujishima A., Honda K. (1972). Electrochemical photolysis of water at a semiconductor electrode. Nature.

[2] Kim W.-T., Choi W.-Y. (2017). Fabrication of TiO_2_ photonic crystal by anodic oxidation and their optical sensing properties. Sensors Actuators A Phys..

[3] Tshabalala Z.P., Swart H.C., Motaung D.E. (2021). Fabrication of TiO_2_ nanofibers based sensors for enhanced CH_4_ performance induced by notable surface area and acid treatment. Vacuum.

[4] Li F., Song H., Yu W., Ma Q., Dong X., Wang J., Liu G. (2020). Electrospun TiO_2_//SnO_2_ Janus nanofibers and its application in ethanol sensing. Mater. Lett..

[5] Kim W.-T., Na K.-H., Lee J.-K., Jang I., Choi D.-S., Choi W.-Y. (2019). Porous TiO_2_ Nanotube Arrays for Drug Loading and Their Elution Sensing. J. Nanosci. Nanotechnol..

[6] Li L., Xie C., Xiao X. (2020). Polydopamine modified TiO_2_ nanotube arrays as a local drug delivery system for ibuprofen. J. Drug Deliv. Sci. Technol..

[7] León A., Reuquen P., Garín C., Segura R., Vargas P., Zapata P., Orihuela P.A. (2017). FTIR and Raman characterization of TiO_2_ nanoparticles coated with polyethylene glycol as carrier for 2-methoxyestradiol. Appl. Sci..

[8] Liu D., Bi Y. (2019). Controllable fabrication of hollow TiO_2_ spheres as sustained release drug carrier. Adv. Powder Technol..

[9] Li H., Wang P., Yi X., Yu H. (2020). Edge-selectively amidated graphene for boosting H_2_-evolution activity of TiO_2_ photocatalyst. Appl. Catal. B Environ..

[10] Khan T.T., Bari G.A.K.M., Kang H.-J., Lee T.-G., Park J.-W., Hwang H.J., Hossain S.M., Mun J.S., Suzuki N., Fujishima A. (2021). Synthesis of N-Doped TiO_2_ for Efficient Photocatalytic Degradation of Atmospheric NO_x_. Catalysts.

[11] Sun X., Li H.-J., Ou N., Lyu B., Gui B., Tian S., Qian D., Wang X., Yang J. (2019). Visible-light driven TiO_2_ photocatalyst coated with graphene quantum dots of tunable nitrogen doping. Molecules.

[12] Blanco M., Monteserín C., Angulo A., Pérez-Márquez A., Maudes J., Murillo N., Aranzabe E., Ruiz-Rubio L., Vilas J.L. (2019). TiO_2_-doped electrospun nanofibrous membrane for photocatalytic water treatment. Polymers.

[13] Liu D., Wang J., Zhou J., Xi Q., Li X., Nie E., Piao X., Sun Z. (2019). Fabricating I doped TiO_2_ photoelectrode for the degradation of diclofenac: Performance and mechanism study. Chem. Eng. J..

[14] Kim K.-P., Kim W.H., Kwon S.M., Kim J.Y., Do Y.S., Woo S. (2021). Enhanced Light Absorption by Facile Patterning of Nano-Grating on Mesoporous TiO_2_ Photoelectrode for Cesium Lead Halide Perovskite Solar Cells. Nanomaterials.

[15] Tsvetkov N., Larina L., Ku Kang J., Shevaleevskiy O. (2020). Sol-gel processed TiO_2_ nanotube photoelectrodes for dye-sensitized solar cells with enhanced photovoltaic performance. Nanomaterials.

[16] Abbas M.M., Rasheed M. (2021). Solid State Reaction Synthesis and Characterization of Cu doped TiO_2_ Nanomaterials. Proceedings of the 2nd International Conference on Materials, Laser science and Applied physics (ICMLAP).

[17] Ren Y., Han Y., Li Z., Liu X., Zhu S., Liang Y., Yeung K.W.K., Wu S. (2020). Ce and Er Co-doped TiO_2_ for rapid bacteria-killing using visible light. Bioact. Mater..

[18] Li R., Yang J., Xu S., Zhou Y., Wang X., Peng H., Du J. (2020). Preparation of Gd-Doped TiO_2_ Nanotube Arrays by Anodization Method and Its Photocatalytic Activity for Methyl Orange Degradation. Catalysts.

[19] Ismail M.A., Hedhili M.N., Anjum D.H., Singaravelu V., Chung S.H. (2021). Synthesis and Characterization of Iron-Doped TiO_2_ Nanoparticles Using Ferrocene from Flame Spray Pyrolysis. Catalysts.

[20] Heng J.Z.X., Tang K.Y., Regulacio M.D., Lin M., Loh X.J., Li Z., Ye E. (2021). Solar-powered photodegradation of pollutant dyes using silver-embedded porous TiO_2_ nanofibers. Nanomaterials.

[21] Tang K.Y., Chen J.X., Legaspi E.D.R., Owh C., Lin M., Tee I.S.Y., Kai D., Loh X.J., Li Z., Regulacio M.D. (2021). Gold-decorated TiO_2_ nanofibrous hybrid for improved solar-driven photocatalytic pollutant degradation. Chemosphere.

[22] Valero-Romero M.J., Santaclara J.G., Oar-Arteta L., van Koppen L., Osadchii D.Y., Gascon J., Kapteijn F. (2019). Photocatalytic properties of TiO_2_ and Fe-doped TiO_2_ prepared by metal organic framework-mediated synthesis. Chem. Eng. J..

[23] Ruggieri F., Di Camillo D., Maccarone L., Santucci S., Lozzi L. (2013). Electrospun Cu-, W- and Fe-doped TiO_2_ nanofibres for photocatalytic degradation of rhodamine 6G. J. Nanoparticle Res..

[24] Li X., Shi J.-L., Hao H., Lang X. (2018). Visible light-induced selective oxidation of alcohols with air by dye-sensitized TiO_2_ photocatalysis. Appl. Catal. B Environ..

[25] Ding H., Xu M., Zhang S., Yu F., Kong K., Shen Z., Hua J. (2020). Organic blue-colored DA-π-A dye-sensitized TiO_2_ for efficient and stable photocatalytic hydrogen evolution under visible/near-infrared-light irradiation. Renew. Energy.

[26] Ahmad I., Kan C. (2017). Visible-light-driven, dye-sensitized TiO_2_ photo-catalyst for self-cleaning cotton fabrics. Coatings.

[27] Li Z.-Q., Mo L.-E., Chen W.-C., Shi X.-Q., Wang N., Hu L.-H., Hayat T., Alsaedi A., Dai S.-Y. (2017). Solvothermal synthesis of hierarchical TiO_2_ microstructures with high crystallinity and superior light scattering for high-performance dye-sensitized solar cells. ACS Appl. Mater. Interfaces.

[28] Zhang G., Zhang S., Wang L., Liu R., Zeng Y., Xia X., Liu Y., Luo S. (2017). Facile synthesis of bird’s nest-like TiO_2_ microstructure with exposed (001) facets for photocatalytic degradation of methylene blue. Appl. Surf. Sci..

[29] Liu H., Zhang L., Li T. (2018). A study of controllable synthesis and formation mechanism on flower-like TiO_2_ with spherical structure. Crystals.

[30] Akshay V.R., Arun B., Mandal G., Mutta G.R., Chanda A., Vasundhara M. (2018). Observation of optical band-gap narrowing and enhanced magnetic moment in co-doped sol–gel-derived anatase TiO_2_ nanocrystals. J. Phys. Chem. C.

[31] Singh J., Sharma S., Sharma S., Singh R.C. (2019). Effect of tungsten doping on structural and optical properties of rutile TiO_2_ and band gap narrowing. Optik.

[32] Hsu J.-C., Lin Y.-H., Wang P.W. (2020). X-ray Photoelectron Spectroscopy Analysis of Nitrogen-Doped TiO_2_ Films Prepared by Reactive-Ion-Beam Sputtering with Various NH_3_/O_2_ Gas Mixture Ratios. Coatings.

[33] Mathew S., Ganguly P., Rhatigan S., Kumaravel V., Byrne C., Hinder S.J., Bartlett J., Nolan M., Pillai S.C. (2018). Cu-doped TiO_2_: Visible light assisted photocatalytic antimicrobial activity. Appl. Sci..

[34] Moradi V., Jun M.B.G., Blackburn A., Herring R.A. (2018). Significant improvement in visible light photocatalytic activity of Fe doped TiO_2_ using an acid treatment process. Appl. Surf. Sci..

[35] Kumaravel V., Rhatigan S., Mathew S., Michel M.C., Bartlett J., Nolan M., Hinder S.J., Gascó A., Ruiz-Palomar C., Hermosilla D. (2020). Mo doped TiO_2_: Impact on oxygen vacancies, anatase phase stability and photocatalytic activity. J. Phys. Mater..

[36] Dong Z., Ding D., Li T., Ning C. (2018). Ni-doped TiO_2_ nanotubes photoanode for enhanced photoelectrochemical water splitting. Appl. Surf. Sci..

[37] Gogoi D., Namdeo A., Golder A.K., Peela N.R. (2020). Ag-doped TiO_2_ photocatalysts with effective charge transfer for highly efficient hydrogen production through water splitting. Int. J. Hydrogen Energy.

[38] Begum T., Gogoi P.K., Bora U. (2017). Photocatalytic degradation of crystal violet dye on the surface of Au doped TiO2 nanoparticle. Indian J. Chem. Technol..

[39] Nguyen C.H., Fu C.-C., Juang R.-S. (2018). Degradation of methylene blue and methyl orange by palladium-doped TiO_2_ photocatalysis for water reuse: Efficiency and degradation pathways. J. Clean. Prod..

[40] Mahy J.G., Cerfontaine V., Poelman D., Devred F., Gaigneaux E.M., Heinrichs B., Lambert S.D. (2018). Highly efficient low-temperature N-doped TiO_2_ catalysts for visible light photocatalytic applications. Materials..

[41] Shao J., Sheng W., Wang M., Li S., Chen J., Zhang Y., Cao S. (2017). In situ synthesis of carbon-doped TiO_2_ single-crystal nanorods with a remarkably photocatalytic efficiency. Appl. Catal. B Environ..

[42] Simsek E.B. (2017). Solvothermal synthesized boron doped TiO_2_ catalysts: Photocatalytic degradation of endocrine disrupting compounds and pharmaceuticals under visible light irradiation. Appl. Catal. B Environ..

[43] Qin D.-D., Wang Q.-H., Chen J., He C.-H., Li Y., Wang C.-H., Quan J.-J., Tao C.-L., Lu X.-Q. (2017). Phosphorus-doped TiO_2_ nanotube arrays for visible-light-driven photoelectrochemical water oxidation. Sustain. Energy Fuels.

[44] Yang C., Shang S., Li X. (2021). Fabrication of sulfur-doped TiO_2_ nanotube array as a conductive interlayer of PbO_2_ anode for efficient electrochemical oxidation of organic pollutants. Sep. Purif. Technol..

[45] Cheng H.-H., Chen S.-S., Yang S.-Y., Liu H.-M., Lin K.-S. (2018). Sol-Gel hydrothermal synthesis and visible light photocatalytic degradation performance of Fe/N codoped TiO_2_ catalysts. Materials..

[46] Liang Y., Sun S., Deng T., Ding H., Chen W., Chen Y. (2018). The preparation of TiO_2_ film by the sol-gel method and evaluation of its self-cleaning property. Materials.

[47] Çırak B.B., Karadeniz S.M., Kılınç T., Caglar B., Ekinci A.E., Yelgin H., Kürekçi M., Çırak Ç. (2017). Synthesis, surface properties, crystal structure and dye sensitized solar cell performance of TiO_2_ nanotube arrays anodized under different voltages. Vacuum.

[48] Kim W.-T., Na K.-H., Park D.-C., Yang W.-H., Choi W.-Y. (2020). Photocatalytic Methylene Blue Degradation of Electrospun Ti–Zn Complex Oxide Nanofibers. Nanomaterials.

[49] Lee C.-G., Na K.-H., Kim W.-T., Park D.-C., Yang W.-H., Choi W.-Y. (2019). TiO_2_/ZnO Nanofibers Prepared by Electrospinning and Their Photocatalytic Degradation of Methylene Blue Compared with TiO_2_ Nanofibers. Appl. Sci..

[50] Wang Y., Tian L., Zhu T., Mei J., Chen Z., Yu D.-G. (2021). Electrospun aspirin/Eudragit/lipid hybrid nanofibers for colon-targeted delivery using an energy-saving process. Chem. Res. Chinese Univ..

[51] Ding Y., Dou C., Chang S., Xie Z., Yu D.-G., Liu Y., Shao J. (2020). Core–shell eudragit s100 nanofibers prepared via triaxial electrospinning to provide a colon-targeted extended drug release. Polymers..

[52] Wang M., Li D., Li J., Li S., Chen Z., Yu D.-G., Liu Z., Guo J.Z. (2020). Electrospun Janus zein–PVP nanofibers provide a two-stage controlled release of poorly water-soluble drugs. Mater. Des..

[53] Wang M., Hou J., Yu D.-G., Li S., Zhu J., Chen Z. (2020). Electrospun tri-layer nanodepots for sustained release of acyclovir. J. Alloys Compd..

[54] Aidana Y., Wang Y., Li J., Chang S., Wang K., Yu D.-G. (2021). Fast Dissolution Electrospun Medicated Nanofibers for Effective Delivery of Poorly Water-Soluble Drugs. Curr. Drug Deliv..

[55] El-Newehy M.H., El-Naggar M.E., Alotaiby S., El-Hamshary H., Moydeen M., Al-Deyab S. (2018). Green electrospining of hydroxypropyl cellulose nanofibres for drug delivery applications. J. Nanosci. Nanotechnol..

[56] Sharaf S., El-Naggar M.E. (2018). Eco-friendly technology for preparation, characterization and promotion of honey bee propolis extract loaded cellulose acetate nanofibers in medical domains. Cellulose.

[57] Abdelgawad A.M., El-Naggar M.E., Hudson S.M., Rojas O.J. (2017). Fabrication and characterization of bactericidal thiol-chitosan and chitosan iodoacetamide nanofibres. Int. J. Biol. Macromol..

[58] El-Naggar M.E., Abdelgawad A.M., Salas C., Rojas O.J. (2016). Curdlan in fibers as carriers of tetracycline hydrochloride: Controlled release and antibacterial activity. Carbohydr. Polym..

[59] El-Newehy M.H., El-Naggar M.E., Alotaiby S., El-Hamshary H., Moydeen M., Al-Deyab S. (2016). Preparation of biocompatible system based on electrospun CMC/PVA nanofibers as controlled release carrier of diclofenac sodium. J. Macromol. Sci. Part A.

[60] Rubin Pedrazzo A., Cecone C., Morandi S., Manzoli M., Bracco P., Zanetti M. (2021). Nanosized SnO_2_ Prepared by Electrospinning: Influence of the Polymer on Both Morphology and Microstructure. Polymers.

[61] Ehrmann A. (2021). Non-Toxic Crosslinking of Electrospun Gelatin Nanofibers for Tissue Engineering and Biomedicine—A Review. Polymers.

[62] Dodero A., Scarfi S., Mirata S., Sionkowska A., Vicini S., Alloisio M., Castellano M. (2021). Effect of crosslinking type on the physical-chemical properties and biocompatibility of chitosan-based electrospun membranes. Polymers.

[63] Chiaradia V., Hanay S.B., Kimmins S.D., de Oliveira D., Araújo P.H.H., Sayer C., Heise A. (2019). Crosslinking of electrospun fibres from unsaturated polyesters by bis-triazolinediones (TAD). Polymers.

[64] Park J.-Y., Lee J.-H., Choi D.-Y., Hwang C.-H., Lee J.-W. (2012). Influence of Fe doping on phase transformation and crystallite growth of electrospun TiO_2_ nanofibers for photocatalytic reaction. Mater. Lett..

[65] Luttrell T., Halpegamage S., Tao J., Kramer A., Sutter E., Batzill M. (2014). Why is anatase a better photocatalyst than rutile - Model studies on epitaxial TiO_2_ films. Sci. Rep..

[66] Günnemann C., Haisch C., Fleisch M., Schneider J., Emeline A.V., Bahnemann D.W. (2019). Insights into Different Photocatalytic Oxidation Activities of Anatase, Brookite, and Rutile Single-Crystal Facets. ACS Catal..

[67] Tayade R.J., Surolia P.K., Kulkarni R.G., Jasra R. (2007). V Photocatalytic degradation of dyes and organic contaminants in water using nanocrystalline anatase and rutile TiO_2_. Sci. Technol. Adv. Mater..

